# Treating Epilepsy with Natural Products: Nonsense or Possibility?

**DOI:** 10.3390/ph16081061

**Published:** 2023-07-26

**Authors:** Milan Malaník, Marie Čulenová, Alice Sychrová, Adrianna Skiba, Krystyna Skalicka-Woźniak, Karel Šmejkal

**Affiliations:** 1 Department of Natural Drugs, Faculty of Pharmacy, Masaryk University, Palackého 1946/1, 61200 Brno, Czech Republic; sychrovaa@pharm.muni.cz (A.S.); smejkalk@pharm.muni.cz (K.Š.); 2 Department of Natural Products Chemistry, Faculty of Pharmacy, Medical University of Lublin, 1 Chodzki Str., 20-093 Lublin, Poland; adrianna.skiba@umlub.pl (A.S.); krystyna.skalicka-wozniak@umlub.pl (K.S.-W.)

**Keywords:** epilepsy, anticonvulsant, natural products, GABA, AMPA, NMDA

## Abstract

Epilepsy is a neurological disease characterized by recurrent seizures that can lead to uncontrollable muscle twitching, changes in sensitivity to sensory perceptions, and disorders of consciousness. Although modern medicine has effective antiepileptic drugs, the need for accessible and cost-effective medication is urgent, and products derived from plants could offer a solution. For this review, we have focused on natural compounds that have shown anticonvulsant activity in in vivo models of epilepsy at relevant doses. In some cases, the effects have been confirmed by clinical data. The results of our search are summarized in tables according to their molecular targets. We have critically evaluated the data we present, identified the most promising therapeutic candidates, and discussed these in the text. Their perspectives are supported by both pharmacokinetic properties and potential interactions. This review is intended to serve as a basis for future research into epilepsy and related disorders.

## 1. Introduction

Epilepsy, one of the most common neurological diseases, affects over 70 million people worldwide [1]. The International League Against Epilepsy (ILAE) offers the following practical clinical definition of epilepsy: (1) at least two unprovoked (or reflex) seizures occurring more than 24 h apart; (2) one unprovoked (or reflex) seizure and a probability of further seizures similar to the general recurrence risk (at least 60%) after two unprovoked seizures, occurring over the next 10 years; and (3) the diagnosis of an epilepsy syndrome [2]. Recurrent seizures significantly worsen quality of life and contribute to epilepsy-related causes of death, such as sudden unexpected death in epilepsy (SUDEP), status epilepticus, accident, drowning, or suicide [1,3]. Although relatively effective pharmacotherapy is currently available, treatment with antiseizure medications is frequently associated with side effects like drowsiness, headaches, or uncontrollable shaking. Furthermore, in some cases, even appropriately chosen antiseizure drugs do not control seizures. This drug-resistant epilepsy is currently treated using surgery or neurostimulatory intervention [1]. It is worth mentioning that mortality caused by epilepsy is generally higher in low-income countries than in high-income countries, probably due to a lack of access to medical facilities and therapy, including antiepileptic drugs (AEDs) [4]. The affordability of AEDs is also a burning question due to the fact that total epilepsy costs were estimated to be USD 119.27 billion per year in 2019 [5]. With populations growing rapidly, especially in developing countries, the search for cost-effective and accessible medicines is like that for the Holy Grail. Additionally, exactly here, plant-derived products could be of priceless assistance as they may offer multiple mechanisms of action, thus overcoming resistance and giving fewer side effects. In addition, they could also be used in combination with conventional drugs to increase their effectiveness. Numerous scientific reports have described the potent anticonvulsant activity of plant extracts and the essential oils of various plants in different animal models of seizures and epilepsy. Unfortunately, a purified form of cannabidiol (CBD, Epidiolex/Epidyolex; >99% CBD) used to treat two rare epilepsies, Lennox–Gastaut syndrome and Dravet syndrome, is the only plant-derived product approved for medical use [6], although many other natural compounds exert significant anticonvulsant activity in vivo. This raises the question of why these active substances have not been further investigated. Can we find more effective anticonvulsants in the plant kingdom than those already used in clinical practice? Could we reduce the total cost of epilepsy using plant-derived products? Moreover, many phytoconstituents possess anti-inflammatory or neuroprotective activity that might also be beneficial. They could, potentially, be administered as adjuvants in the therapy of epilepsy. Although the antiepileptic activity of natural compounds has been reviewed several times from different points of view, we have focused more on the most perspective phytoconstituents to point out their pharmacokinetic properties and where it is possible, the structure–activity relationships of various groups of compounds are also mentioned. We have therefore summarized the characteristics of potent natural anticonvulsants in this review in order to arouse interest, consider their use in clinical trials, and evaluate their potential use in clinical practice.

## 2. Data Collection Process

This review results from an in-depth search for scientific papers containing the terms “epilepsy”, “antiepileptic”, “anticonvulsant”, “antiseizure”, and “seizure”, alone and subsequently also their combinations with the keywords “natural compounds”, “natural products”, “flavonoid”, “terpene”, “terpenoid”, “coumarin”, “phenolic”, or “alkaloid” using Boolean operator AND. The scientific databases Science Direct, Web of Science, and Google Scholar were used to collect scientific papers, as was the US database clinicaltrials.gov, which deals with ongoing clinical trials. Only pure and structurally characterized compounds have been included in this review. Plant extracts, essential oils, and mixtures of compounds were excluded as these were often not well-defined, and it is difficult to determine whether the components would be active alone or synergy plays a crucial role which hinders their potential standardization. We included in the text only compounds affecting molecular targets and excluded those possessing antioxidant and anti-inflammatory properties as this topic is large enough for a separate review. The anticonvulsant activity of many natural substances has been reported at doses too high to be applicable in clinical practice, and we therefore include only compounds reported to be active at doses equal to or lower than 100 mg/kg, which represents the application of a maximum of 7 g of a drug in an average 70 kg person.

## 3. Conventional Therapy

Modern pharmacotherapy of epilepsy is based on an individual-patient-oriented approach. The choice of AED is therefore based on the conditions of the individual patient, including demographic and physiological conditions, comorbidities, and psychosocial environment. Generally, monotherapy with the most appropriate drug is started. Polytherapy is initiated only after three drugs, tried individually, have failed. Naturally, AEDs with diverse mechanisms of action are usually combined in the hope of achieving possible synergistic effects [7].

AEDs have traditionally been categorized based on their mode of action and especially the frequency and seriousness of any adverse effects. The first-generation drugs affect mostly the voltage-gated Na^+^ channels and escalate GABAergic transmission. Modern AEDs exhibit distinct mechanisms of action directed to specific receptors or specific receptor subunits. These unique mechanisms eliminate most of the side effects that occurred with the older generation AEDs. Based on their mechanism of action, we can divide AEDs into three main groups: (1) AEDs that reduce the presynaptic excitability and release of neurotransmitters by affecting voltage-gated Na^+^ channels (e.g., carbamazepine, phenytoin) and Ca^2+^ channels (e.g., gabapentin, pregabalin) and acting on presynaptic vesicular SV2A protein (e.g., levetiracetam, brivaracetam); (2) AEDs that potentiate GABAergic transmission by prolonging or increasing the frequency of opening of the chloride channel of the GABA_A_ receptor, or by inhibiting the degradation or transport of GABA from the synapse (e.g., clonazepam, phenobarbital, tiagabine, vigabatrin); and (3) AEDs that reduce the postsynaptic excitability by affecting AMPA or NMDA receptors (e.g., perampanel). Some AEDs have multiple mechanisms of action (e.g., valproate, lamotrigine, topiramate), and for others, the exact mode of action is not fully understood [8,9].

New drugs are emerging. Although none provide better anticonvulsant potency than conventional therapy, they usually have fewer side effects and better pharmacokinetic profiles. Older generation AEDs (e.g., phenobarbital, primidone, and benzodiazepines) are characterized by a sedative effect ranging from mild drowsiness or tiredness to profound lethargy; severe idiosyncratic reactions may sometimes occur (e.g., carbamazepine, phenytoin), whereas modern AEDs are often associated with adverse psychiatric effects (e.g., topiramate, vigabatrin, zonisamide) [10]. As can be seen, no AEDs without adverse effects are known to exist, and the hunt for new medications should therefore be directed towards natural products which do not frequently exert adverse effects.

Studies available to date suggest that natural products show mechanisms of action similar to those of clinically used drugs. Some may also have multiple effects, including neuroprotective and anti-inflammatory activity. This complex activity along with having fewer side effects and interactions represents the main advantage of natural anticonvulsant drugs.

## 4. Molecular Targets of Natural Products and Experimental Models of Epilepsy

**Figure 1:**
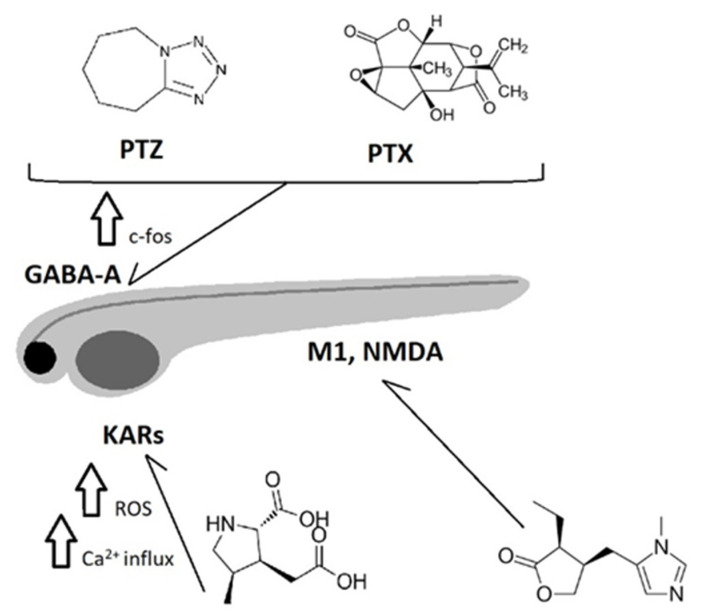
Seizure-inducing agents and proposed mechanisms of action. PTZ: pentylenetetrazole; PTX: picrotoxin; GABA-A: γ-aminobutyric acid-A receptor; KARs: kainate receptors; M1: muscarinic receptor M1; NMDA: N-methyl-D-aspartate; ROS: reactive oxygen species; up arrow: increase.

As mentioned above, the therapy of epilepsy involves a complex approach, and various AEDs affect distinct molecular targets. The same is true for natural substances. Based on the selected experimental model and seizure-inducing agent, we can predict the target protein (Figure 1). Currently, several animal models have become well-established for evaluating the anticonvulsant activity of plant-derived compounds.

Antiepileptic activity is tested primarily on mice and rats, where the inhibition of convulsions can be observed according to the seizure-inducing agent used. Neurons can also be removed from epileptic rodents and histopathological alterations studied ex vivo.

Several alternatives have been introduced into epilepsy research to replace mammalian models of seizures. The simplicity of the roundworm *Caenorhabditis elegans* enables it to serve as a basic model for understanding how genes specify the development of the nervous system. However, its small neural circuit limits comparison with humans [11,12]. Other non-mammalian models used in the study of epilepsy are *Xenopus laevis* oocytes and tadpoles. *Xenopus* oocytes represent a valuable tool to identify the types of ligand-gated ion channels present in different neuronal systems. Proteins can rapidly and easily be expressed and analyzed; thus, oocyte assays are predominantly used to identify GABA_A_ receptor subunits influenced by substances under study. However, the *Xenopus* oocytes model has disadvantages. Its membrane and intracellular organization differs from that of mammalian neurons and the optimum temperature (18–22 °C) maintained during the analysis of oocytes is approximately half the normal mammalian body temperature [12].

The zebrafish *Danio rerio* is currently widely recommended for testing neurobiological disorders because its genes are similar to human genes. Zebrafish have homologs for at least 85% of the recognized epilepsy genes found in humans. Furthermore, the larvae of zebrafish can develop rapidly ex utero, the fish are very fecund, and convulsant chemicals can easily be added directly into the water in which they swim. Together these features create a very powerful experimental model [11,12].

Seizures are commonly induced by various chemoconvulsants, but preliminary testing, performed by using the maximal electroshock (MES) test, has led to the discovery of many clinically effective AEDs. The MES test is a reasonable model of grand mal seizures (i.e., generalized tonic-clonic seizures) and was once considered to be nonselective with respect to molecular targets and mechanisms of action; however, nowadays, it is thought to be sensitive to drugs that block Na^+^ channels. In any case, because the MES test can produce false positive data, it is no longer recommended for any purpose [13]. Pentylenetetrazole (PTZ), the chemoconvulsant most often used to induce acute seizures acts as a GABA_A_ antagonist. That the subcutaneous PTZ test is therefore most sensitive to GABA mimetic drugs limits its applicability because compounds with other mechanisms of action could be missed [14]. In addition to PTZ, other chemical GABA_A_ antagonists, such as picrotoxin (PTX) or bicuculline, possess the ability to induce absence-like seizures [12]. Kainic acid (a glutamate analog) and pilocarpine (a muscarinic agonist) are used as experimental models of status epilepticus or to produce chronic epilepsy with recurrent spontaneous seizures. Chronic models of epilepsy are used to predict clinical efficacy and adverse effects [15].

Aside from chemical models of epilepsy, genetic zebrafish models play an irreplaceable role in evaluating anticonvulsant activity. Genetic models are based on knocking out the genes responsible for an epilepsy-related disorder. Here, we briefly describe some of them. *Kcnj10a* is a model responsible for EAST syndrome (epilepsy, ataxia, sensorineural deafness, and renal tubulopathy), which causes seizures in infancy [16]. The *Scn1Lab* gene encodes the voltage-gated sodium channel Na_v_1.1, the disruption of which causes Dravet syndrome [17]. *Stxbp1* (gene encoding syntaxin-binding protein 1) is a model for EIEE (early infantile epileptic encephalopathy), that is, childhood epilepsy. It plays a role in epilepsy and metabolic, physiological, and behavioral changes. Mutations of this protein are found in Dravet syndrome and Lennox–Gastaut syndrome [18]. *Gabra1* encodes GABA type A receptor subunit alpha 1. The knockdown of this gene revealed a reduced amount of GABA_A_ receptors in the brain, which causes epilepsy in juvenile zebrafish, and probably not only causes an imbalance between neuronal inhibition and excitation but also disrupts early development of the brain [19]. *Stx1b*—mutations in this gene cause epilepsy syndromes associated with fever. These seizures manifest ranging from febrile seizures to severe epileptiform encephalopathies. In zebrafish, it manifests as seizure-like behavior accompanied by epileptiform discharges resulting from hyperthermia [20].

Furthermore, an alteration in the expression of an epilepsy-related gene has also been observed when different methods of inducing convulsions were applied. Brain-derived neurotrophic factor (BDNF) plays an important role in epileptogenesis because its level is dramatically increased in both animal models and humans with epilepsy. The ability to regulate the neuronal morphology linked with neuroprotective effects is attributed to BDNF; it also reduces the excitability of neurons and prevents or slows seizures and seizure-induced neuronal damage. On the other hand, seizures induce the expression of BDNF, which then activates its receptor TrkB, and the consequent cascade can produce structural plasticities of the hippocampal dentate granule cells similar to those identified in the epileptic brain [21,22]. Glutamic acid decarboxylase (GAD) is the rate-limiting enzyme for the synthesis of GABA. Its isoform GAD65 is related to epilepsy, and the family of these enzymes represents a promising target not only for potential antiepileptic drugs but also in the therapy of various autoimmune diseases [23,24]. The protooncogene *c-fos* is upregulated in response to such stress stimuli as seizures; therefore, natural compounds that reduce the expression of *c-fos* in experimental models are considered to be potential anticonvulsants [25]. In addition, many plant-derived products have been identified as neuroprotectants that can be used in the treatment of epilepsy. Examples, such as cytokines (TNF-α, IL-1β, IL-6) or apoptotic proteins (Bax/Bcl-2), most often affect various signaling molecules linked to neurotoxicity and inflammation in the brain [26].

Modeling the different types of epilepsy that parallel human-type enabled the creation of a platform for the high-throughput screening of drugs that might be used to treat those epilepsies. Using this platform is not as complicated and time-consuming as would be the case for rodent models, so it might be perfect for generating new epilepsy models to choose drugs for further examination.

**Figure 2:**
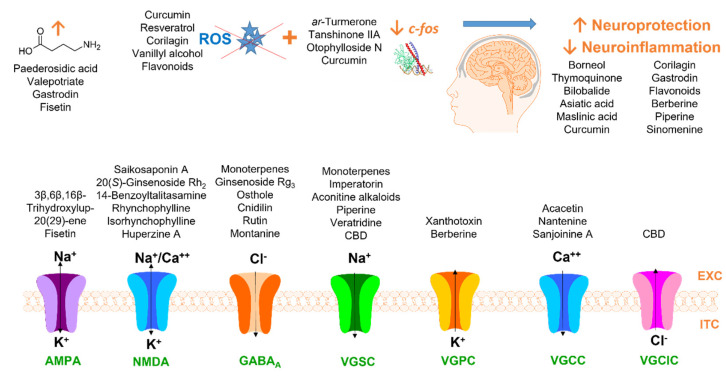
Natural products and their proposed targets of anticonvulsant activity. ROS: reactive oxygen species; CBD: cannabidiol; EXC: extracellular; ITC: intracellular; AMPA: α-amino-3-hydroxy-5-methylisoxazole-4-propionic acid; NMDA: N-methyl-D-aspartate; GABA_A_: γ-aminobutyric acid_A_; VGSC: voltage-gated sodium channel; VGPC: voltage-gated potassium channel; VGCC: voltage-gated calcium channel; VGClC: voltage-gated chloride channel.

The small molecules isolated from plants have been summarized in Figure 2. They differ in their target structures, but all of them can influence the aforementioned proteins. We have therefore summarized the results of our search in tables that describe the mechanism of action according to the molecular target. The tables include the names of the compounds and their classification, the effective doses, animal models used, seizure-inducing agents, and proposed mechanisms of action. Natural anticonvulsants were administered as a treatment for several days (mentioned in tables in brackets). If this is not specified in the tables, each test substance was administered 10–90 min prior to the injection of the seizure-inducing agent. We describe in detail the interaction of phytoconstituents with voltage-gated ion channels (Table 1), GABA (Table 2), and AMPA and NMDA receptors (Table 3). Those natural products that affect diverse targets and thus relieve seizures are summarized in Table S1. Even more compounds exhibit anticonvulsant activity through mechanisms that are still unknown. These are identified in Table S2, but only those considered relevant receive mention in the text. Neuroprotective natural products are summarized in Table S3. We did not pay increased attention to the detailed description of neuroprotection in the text as this area has been reviewed extensively, e.g., by Brahmachari [26]. Furthermore, we discuss in the text only those natural compounds present in plants in significant amounts that could serve as a valuable source for effective isolation with considerable yields.

### 4.1. Natural Products That Affect Voltage-Gated Na^+^ and Ca^2+^ Channels

Voltage-gated sodium channels (VGSCs) initiate and conduct action potentials in excitable cells such as neurons or muscle cells, whereas voltage-gated calcium channels (VGCCs) are activated during action potentials, and they conduct the influx of Ca^2+^ into cells to initiate physiological processes, such as neurotransmission and muscle contraction [27]. Both channels represent molecular targets for drugs used in the treatment of epilepsy (see above).

Not many plant-derived compounds that affect VGSCs and VGCCs have been reported (Table 1). The effects of monoterpenes on various ion channels have been reviewed recently by Oz et al. [28], but most of the compounds mentioned exerted anticonvulsant effects only at doses too high to be implemented in clinical practice. Therefore, alkaloids and coumarins seem to have better prospects than monoterpenoids.

Several *Aconitum* alkaloids have been proven to interact with Na^+^ channels [29,30,31,32,33,34]. Unfortunately, these alkaloids have been isolated only from *Aconitum* species endemic to some regions of China. Furthermore, the isolation of these compounds is very tricky, with small yields. Because the total synthesis of aconitine-type alkaloids remains elusive, the prospects for *Aconitum* alkaloids are vague.

On the other hand, piperine, the major bioactive component in black pepper (*Piper nigrum*), significantly inhibited Na^+^ channel activity in mice [35]. Moreover, co-administration with carbamazepine (CBZ) or phenytoin decreased the elimination of these AEDs and enhanced their bioavailability [36]. The metabolism of piperine in the liver is limited, and its high blood–brain barrier permeability has been demonstrated in the Caco-2 monolayer model [37]. Its only limiting factor is poor solubility in water, and this can be improved, e.g., by a nanoprecipitation method leading to enhanced oral bioavailability and brain delivery of piperine after oral administration [38]. Altogether, piperine represents a very promising candidate for further evaluation in clinical trials, albeit with the caveat that increased attention must be paid to piperine-mediated drug interactions [39].

Similarly, the co-administration of coumarins significantly reduced the ED_50_ values of AEDs in the MES test in mice, as observed for imperatorin (40 mg/kg, i.p.) in combination with CBZ, phenobarbital, or phenytoin [40], and for xanthotoxin (50 and 100 mg/kg, i.p.) in combination with CBZ and valproate (VPA), respectively [41]. Additionally, xanthotoxin increased the total brain concentration of CBZ and VPA by about 84% and 46%, respectively, probably by inhibiting P-glycoprotein [41]. Therefore, co-administering natural compounds with conventional AEDs could be a way to increase the anticonvulsant activity of AEDs and thus improve the comfort of patients suffering from epilepsy.

**Table 1:** Natural products that affect VGSCs, VGCCs, or VGPCs.

Compound	Effective Dose	Animal Model	Seizure-Inducing Agent	Mechanism	Source
Paeoniflorin (monoterpene)	100 mg/kg/day, p.o. (for 10 days)	Male immature Lewis rats	Hyperthermia	Suppression of [Ca^2+^]_i_ elevation via mGluR5	[42]
Thymol (monoterpene)	50 and 100 mg/kg, i.p.	Male Swiss mice Male Wistar rats	PTZ, MES, Strychnine, 4-AP	Possibly via positive modulation of GABA_A_ and voltage-dependent Na^+^ channel blockade	[43]
Iritectol G (triterpene)	10 μM	Neocortical neurons of C57BL/6 mice	4-AP	Interaction with inactivated state of VGSC	[44]
Imperatorin (coumarin)	30–50 µM	NG108-15 cells	Voltage-clamp assay	Inhibition of VGSC	[45]
Xanthotoxin (coumarin)	50 and 100 mg/kg, i.p. + CBZ 100 mg/kg, i.p. + VPA	Male Swiss mice	MES	Inhibition of P-glycoprotein Inhibition of VGPC Modulation of calcium-dependent potassium channels	[46]
Acacetin (flavonoid)	10 and 50 mg/kg, i.p.	Male Sprague Dawley rats	KA	Inhibition of glutamate release by decrease in voltage-dependent Ca^2+^ entry	[47]
Aconitine (alkaloid)	1 μM	Hippocampal slices of male Wistar rats	Low Mg^2+^-ACSF	Modulation of Na^+^ channels	[29]
3-Acetylaconitine (alkaloid)	0.01–1 μM	Hippocampal slices of male Wistar rats	Mg^2+^-free ACSF Bicuculline	Inactivation of Na^+^ channels	[31]
Lappaconitine (alkaloid)	1–100 μM	Hippocampal slices of male Wistar rats	Low Mg^2+^-ACSF Bicuculline	Blockade of Na^+^ channels	[32]
N-desacetyl lappaconitine (alkaloid)	1–100 μM	Hippocampal slices of male Wistar rats	Low Mg^2+^-ACSF Bicuculline	Blockade of Na^+^ channels	[32]
1-Benzoylnapelline (alkaloid)	1–100 μM	Hippocampal slices of male Wistar rats	Low Mg^2+^-ACSF Bicuculline	Modulation of Na^+^ channels	[33]
6-Benzoylheteratisine (alkaloid)	0.01–10 μM	Hippocampal slices of male Wistar rats	Bicuculline	Blockade of Na^+^ channels	[34]
Nantenine (alkaloid)	20–50 mg/kg, i.p.	Male albino mice	PTZ MES	Decrease in Ca^2+^-influx into the cell	[48]
Piperine (alkaloid)	5, 10, and 20 mg/kg, i.p.	Male Swiss mice	PTZ, MES, NMDA, PTX, Bicuculline, BAYK-8644, Strychnine	Na^+^ channel antagonist activity	[35]
Veratridine (alkaloid)	1 μM	Hippocampal slices of male Wistar rats	Low Ca^2+^/high Mg^2+^-ACSF	Block of inactivation of Na^+^ channels	[30]

### 4.2. Natural Products That Affect the Gabaergic Transmission

γ-Aminobutyric acid (GABA) is the main inhibitory neurotransmitter in the central nervous system (CNS). It is synthesized from glutamate by glutamic acid decarboxylase (GAD). GABA receptors can be divided into the ionotropic GABA_A_ receptors and the metabotropic GABA_B_ receptors. GABA_A_ receptors are ligand-gated ion channels that increase the flow of chloride ions into the cell and thus promote inhibitory effects in the brain [49]. The potentiation of GABAergic transmission is one of the main targets of the AEDs currently used in clinical practice (see above).

Whereas only a few natural products interact with VGSCs and VGCCs, phytoconstituents most often affect the modulation of GABA_A_ receptors (Table 2). The most significant anticonvulsant effects mediated by GABAergic transmission have been observed for terpenoids. Manayi et al. [50] have reviewed the ability of natural terpenoids to modulate the GABAergic system, but most of the compounds identified exerted anticonvulsant effects only at doses too high to be implemented in clinical practice. We have therefore pointed out only those terpenes that exhibit notable activity at relevant doses.

Monoterpenes and sesquiterpenes are constituents of essential oils, and they have properties plausible for a potential application. Their low molecular weight and high lipophilicity allow them to penetrate membranes and interact with epilepsy-related proteins [51]. Thymoquinone, the dominant compound in black seed oil (*Nigella sativa*), has shown the most notable activity of all the monoterpenes found in this search, inhibiting convulsions in male BALB/c mice at the relatively low dose of 40 mg/kg [52]. Preclinical findings led to a pilot study in children with refractory epilepsy (see Section 5). The results demonstrated that thymoquinone was effective and well-tolerated [53]. Moreover, thymoquinone potentiated the antiepileptic properties of VPA [50] and phenytoin [54]. It also displayed a neuroprotective effect in Sprague Dawley rats by phosphorylating cAMP response element-binding protein (CREB) [55] and downregulating TNF-α and COX-2 [56]. Unfortunately, thymoquinone inhibits the activity of cytochrome P450 2C9 (CYP2C9), and this must be taken into account when thymoquinone is co-administered with phenytoin [57]. To sum up, its anticonvulsant and neuroprotective properties make thymoquinone a very promising substance that deserves further investigation with emphasis on an in-depth exploration of its pharmacokinetics and potential interactions.

Iridoids seem to be effective in the inhibition of convulsions as well, and especially the reports of the anticonvulsant activity of the extracts of *Valeriana* species are increasingly frequent. Pretreatment with valepotriate (5, 10, 20 mg/kg/day, i.p.) protected mice against MES and PTZ-induced convulsions, but it was far less effective than diazepam (4 mg/kg/day, i.p.) [58]. Significant anticonvulsant activity has also been reported for paederosidic acid, a rare sulfur-containing iridoid [59], but such compounds are, unfortunately, very unstable under acidic conditions, which makes their peroral administration difficult [60].

Bilobalide, the main sesquiterpene trilactone found in the leaves of *Ginkgo biloba*, must be included. Bilobalide (30 mg/kg, p.o., once a day for 4 days) elevated GABA levels in the hippocampus, cerebral cortex, and striatum of male ddY mice, possibly through the potentiation of the activity of GAD [61]. Also considering its neuroprotective effect [62], makes bilobalide seems very promising, although Ng et al. point out that bilobalide negatively modulated the action of GABA at α_1_β_2_γ_2L_ GABA_A_ receptors [63].

An even more promising natural product affecting GABA transmission has been found in huperzine A (HupA), a dietary supplement used in the USA as a memory enhancer. HupA is an acetylcholinesterase inhibitor isolated from the Chinese club moss *Huperzia serrata*. It also exerts anti-inflammatory and neuroprotective effects by activating nicotinic cholinergic receptors [64]. In addition, HupA has delivered seizure relief in a 6 Hz model, with an ED_50_ value of 0.34 mg/kg in the 32 mA paradigm, being 57 times more potent than levetiracetam and 301 times more potent than VPA [65]. It also suppressed PTZ-induced seizures in rats by activating the cortical transmission of GABA [66]. These findings, together with the favorable pharmacokinetic properties of HupA in humans [67], have led to clinical testing (see Section 5).

Recently, several coumarins have been reported to effectively inhibit PTZ-induced seizures in the zebrafish larvae model of epilepsy at doses lower than those of the positive controls diazepam (10 mM) and VPA (1 mM), respectively [68,69]. Based on the seizure-inducing agent used (PTZ), it was postulated that the test coumarins interfered with the GABA transmission. Coumarins have previously been shown to inhibit the activity of GABA transaminase (GABA-T), the main degradative enzyme of GABA [70]. This hypothesis was supported by a molecular docking study of oxypeucedanin hydrate, the most active furanocoumarin, to the structural model of GABA-T. The results indicated that a bulky substituent at the C5 position is crucial for antiseizure activity, whereas an analogous bulky moiety substituted at the C8 position diminishes the activity [69]. Similar results have been reported by Singhuber et al. [71] in a study dealing with the modulation of GABA-induced chloride currents by selected coumarin derivatives on recombinant α_1_β_2_γ_2S_ GABA_A_ receptors expressed in *Xenopus laevis* oocytes [71]. On the other hand, exactly the opposite results were found for the mice MES test. C5-substituted furanocoumarins were inactive, whereas C8-substituted furanocoumarins exerted strong anticonvulsant activity [72]. Hence, more studies are needed to clarify the structure–activity relationship with respect to the model of epilepsy used as well as to find out more about bioavailability. However, as coumarins are simple molecules, they are ideal for chemical synthesis and modifications to improve their pharmacodynamic and pharmacokinetic properties.

**Table 2:** Natural products with influence on GABAergic transmission.

Compound	Effective Dose	Animal Model	Seizure-Inducing Agent	Mechanism	Source
Thymol (monoterpene)	50 and 100 mg/kg, i.p.	Male Swiss mice Male Wistar rats	PTZ, MES, Strychnine, 4-AP	Possibly via positive modulation of GABA_A_ and voltage-dependent Na^+^ channel blockade	[43]
Thymoquinone (monoterpene)	40 mg/kg/day, p.o. (for 7 days)	Sprague Dawley rats	PTZ	Activation of GABA_B1_R/CaMKII/_p_CREB pathway	[55]
Paederosidic acid (iridoid)	5–40 mg/kg, i.p.	Male ICR mice Sprague Dawley rats	MES PTZ	Upregulation of GAD65	[59]
Valepotriate (iridoid)	5–20 mg/kg/day, i.p. (for 3 weeks)	Male ICR mice Sprague Dawley rats	MES PTZ	Upregulation of GABA_A_, GAD65, and Bcl-2 and downregulation of caspase-3	[58]
Bilobalide (sesquiterpene)	30 mg/kg/day, p.o. (for 4 days)	Hippocampus, cortex, and striatum of male ddY mice	INH	Elevation of GABA levels Potentiation of GAD activity	[61]
Curcumol (sesquiterpene)	100 mg/kg/day, i.p. (for 3 days)	Male C57BL/6J mice	PTZ KA	Facilitation of GABAergic inhibition	[73]
(+)-Dehydrofukinone (sesquiterpene)	10, 30, and 100 mg/kg, i.p.	Female Swiss mice	PTZ	Modulation of GABA_A_ receptors	[74]
Betulin (triterpene)	50 and 100 mg/kg, i.p.	Male ICR mice	Bicuculline	Binding to the GABA_A_ receptor	[75]
Ginsenoside Rg_3_ (triterpene)	100 μM	*Xenopus laevis* oocytes	Electrode voltage-clamp technique	GABA_A_ receptor activation via interaction with the γ_2_ subunit	[76]
Ursolic acid stearoyl glucoside (triterpene)	50 mg/kg, i.p.	Wistar albino rats	MES INH	Possibly via GABA receptor stimulation	[77]
Embelin (benzoquinone)	0.156–0.625 mg/kg, i.p.	Adult zebrafish	PTZ	Affinity toward GABA_A_ receptor	[78]
Cnidilin (coumarin)	300 µM	*Xenopus* oocytes	Two-microelectrode voltage clamp assay	Modulation of GABA_A_ receptors of the subunit combination α_1_β_2_γ_2S_	[79]
Osthole (coumarin)	300 µM	*Xenopus* oocytes	Two-microelectrode voltage clamp assay	Modulation of GABA_A_ receptors of the subunit combination α_1_β_2_γ_2S_	[79]
Lucidafuranocouma-rin A (coumarin)	10–16 μM	Zebrafish larvae	PTZ	Possibly via interaction with the GABA_A_ receptor	[68]
Oxypeucedanin (coumarin)	10–40 μM	Zebrafish larvae	PTZ	Possibly via interaction with the GABA_A_ receptor	[69]
Oxypeucedanin hydrate (coumarin)	20–50 μM	Zebrafish larvae	PTZ	Possibly via interaction with the GABA_A_ receptor	[69]
Notopterol (coumarin)	0.25–2 μM	Zebrafish larvae	PTZ	Possibly via interaction with the GABA_A_ receptor	[69]
Pimpinellin (coumarin)	20–80 μM	Zebrafish larvae	PTZ	Possibly via interaction with the GABA_A_ receptor	[69]
Hyuganin C (coumarin)	2.5–20 μM	Zebrafish larvae	PTZ	Possibly via interaction with the GABA_A_ receptor	[69]
Rosmarinic acid (phenolic)	30 mg/kg, i.p.	Female C57BL/6 mice	PTZ Pilocarpine	Probably activation of the GABAergic system	[80]
Chlorogenic acid (phenolic)	5 mg/kg/day, p.o. (for 15 days)	Male Swiss albino mice	Pilocarpine	Suppressing glutamate receptors, neuroprotective effect	[81]
Gastrodin (phenolic)	60 mg/kg/day, p.o. (for 7 days)	Mongolian gerbils	Genetic seizure model (seizure-sensitive gerbils)	Decrease in GABA degradation Decrease in GABA-T, SSADH, and SSAR immunoreactivities	[82]
Rutin (flavonoid)	50 and 150 nM, i.c.v.	Male Wistar rats	PTZ	Positive allosteric modulation of the GABA_A_ receptor complex via interaction at the benzodiazepine site	[83]
Wogonin (flavonoid)	5 and 10 mg/kg, i.p.	Male Sprague Dawley rats	MES PTZ	Potentiation of the activity of GABA	[84]
Vitexin (flavonoid)	100 and 200 µM, i.c.v.	Male Wistar rats	PTZ	Interaction with GABA_A_ benzodiazepine receptor complex	[85]
	10 mg/kg/day, p.o. (for 15 days)	Male Swiss albino mice	Pilocarpine	Suppressing glutamate receptors, neuroprotective effect	[81]
Nobiletin (flavonoid)	12.5, 25, and 50 mg/kg/day, o.g. (for 6 days)	C57BL/6 mice	PTZ	Modulation GAD65/GABA_A_ expression, BDNF-TrkB, PI3K/Akt	[86]
(+)-Erythravine (alkaloid)	0.25–3 μg/μL, i.c.v.	Male Wistar rats	Bicuculline, NMDA, KA, PTZ	Probably modifying GABA neurotransmission	[87]
(+)-11-α-Hydroxy-erythravine (alkaloid)	0.25–3 μg/μL, i.c.v.	Male Wistar rats	Bicuculline, NMDA, KA, PTZ	Probably modifying GABA neurotransmission	[87]
Huperzine A (alkaloid)	0.6 mg/kg, i.p.	Male Sprague Dawley rats	PTZ	Activation of cortical GABA transmission	[66]
Lobeline (alkaloid)	10, 20, 30 mg/kg, i.p.	Male Swiss mice	PTZ Strychnine	Enhancing the GABA release	[88]
Montanine (alkaloid)	30 and 60 mg/kg, i.p.	Swiss mice and Wistar rats of either sex	PTZ	Modulation of several neurotransmitter receptor systems including GABA_A_ receptors	[89]
Piperine (alkaloid)	2.5, 5, 10, and 20 mg/kg, i.p.	Male Swiss mice	Pilocarpine	Multiple anticonvulsant mechanisms, modulation of the GABA system, antioxidant, and anti-inflammatory activity	[90]

### 4.3. Natural Products That Reduce Postsynaptic Excitability by Affecting AMPA or NMDA Receptors

Glutamate is the principal excitatory neurotransmitter in the brain, and therefore, glutamate receptor agonists, such as α-amino-3-hydroxy-5-methylisoxazole-4-propionic acid (AMPA), N-methyl-D-aspartate (NMDA), and kainate, act as elicitors of seizures. Glutamate receptors can be divided into ionotropic glutamate receptors (ligand-gated ion channels) and metabotropic glutamate receptors (G-protein-coupled receptors) [91]. Among the ionotropic glutamate receptors, the AMPA-type and NMDA-type glutamate receptors are the most important, as certain AEDs affect these ionotropic receptors (e.g., perampanel and topiramate).

Interestingly, no reports of natural products interacting with AMPA receptors have been found during our search, except for one recent study describing the potent anticonvulsant activity of magnolol and honokiol in a model of therapy-resistant epilepsy. However, the authors postulate the involvement of not only AMPA receptors but also GABA_A_ and cannabinoid receptors [92]. Magnolol and honokiol, the main bioactive substances in the bark of *Magnolia officinalis*, are known for antioxidant, anti-inflammatory, and neuroprotective properties that make them promising for further research in the field of epilepsy, especially at a time when knowledge of their toxicity and bioavailability is accumulating [93].

*Panax ginseng* is well-known for its neuroprotective properties with ginsenosides as the main active constituents. Among the test ginsenosides, 20(*S*)-Rg_3_ and 20(*S*)-Rh_2_ inhibited NMDA receptors. However, 20(*S*)-Rg_3_ interacted with the glycine site, while 20(*S*)-Rh_2_ likely did so with the polyamine-binding site of NMDA receptors. (*R*)-isomers were inactive and the mono-glycosylated moiety at C-3 was found to be essential for binding to polyamine sites [94]. Further assays showed that 20(*S*)-Rg_3_ regulated GABA_A_ receptor activity by interacting with the γ_2_ subunit [76], demonstrating its multitarget mechanism of action. Nevertheless, the bioavailability of ginsenosides after oral administration is relatively poor. Low water solubility and easy degradation by gastric acid and gut microbiota are the crucial disadvantages. Therefore, their absorption needs to be enhanced by sophisticated formulation strategies [95].

**Table 3:** Natural products that affect AMPA or NMDA receptors.

Compound	Effective Dose	Animal Model	Seizure-Inducing Agent	Mechanism	Source
20(*S*)-Ginsenoside Rh_2_ (triterpene)	10 μM	Hippocampal neurons of Sprague Dawley rats	NMDA, AMPA, KA, Glycine	Inhibition of NMDA receptors via the interaction with the polyamine-binding site	[94]
Saikosaponin A (triterpene)	1 μM	Hippocampal neurons of Sprague Dawley rats	Kynurenic acid PTX	Inhibition of NMDA receptor current and persistent sodium current (*I_NaP_*)	[96]
3β,6β,16β-Trihydroxylup-20(29)-ene (triterpene)	30 mg/kg, i.g.	Swiss mice	PTZ	Possibly via Na^+^, K^+^-ATPase activity maintenance	[97]
6-Gingerol (phenolic)	37.5 μM	Zebrafish larvae	PTZ	Inhibition of NMDA receptors via the interaction with the glutamate-binding site	[98]
Magnolol (neolignan)	12.5 µM 30 mg/kg, i.p.	Adult zebrafish Male NMRI mice	PTZ, EKP 6-Hz test	Probably targeting GABA_A_, cannabinoid, and AMPA receptors	[92]
Honokiol (neolignan)	6.25 µM	Adult zebrafish	PTZ EKP	Probably targeting GABA_A_, cannabinoid, and AMPA receptors	[92]
Huperzine A (alkaloid)	1, 2, and 3 mg/kg, i.m.	Male Sprague Dawley rats	NMDA	NMDA antagonism	[99]
14-Benzoyltalitasamine (alkaloid)	0.3–10 μM	Hippocampal slices of male Wistar rats	Low Mg^2+/^high K^+^-ACSF	Modulation NMDA receptors	[100]
Ibogaine (alkaloid)	ED_50_ 31 mg/kg, i.p.	Male NIH Swiss mice	MES NMDA	Blockade of NMDA receptors	[101]
Rhynchophylline (alkaloid)	30 μM	*Xenopus laevis* oocytes injected with total RNA from Male Wistar rat cortices or cerebelli	NMDA Glycine	Noncompetitive antagonist of the NMDA receptor	[102]
	100 μM, i.c.v.	Male Sprague Dawley rats	Pilocarpine	Inhibition of the persistent sodium current *I_NaP_* and NMDA receptor current	[103]
Isorhynchophylline (alkaloid)	30 μM	*Xenopus laevis* oocytes injected with total RNA from Male Wistar rat cortices or cerebelli	NMDA Glycine	Noncompetitive antagonist of the NMDA receptor	[102]

### 4.4. Natural Products with Multiple Mechanisms of Action: Cannabinoids

*Cannabis sativa* contains more than 100 compounds (lipophilic phytocannabinoids) with different therapeutic potentials and because phytocannabinoids affect diverse epilepsy-related targets [104], they have been given a separate section. Medicinal marijuana is applicable as a treatment option mainly for patients with chronic, autoimmune, inflammatory, degenerative, or oncological illnesses, and also for palliative care [105]. Multiple in vitro and in vivo preclinical trials have reported antiepileptic effects for several constituents of medical marijuana. Compounds of interest include psychoactive ∆^9^-tetrahydrocannabinol (∆^9^-THC) and structurally similar cannabidiol (CBD), along with non-psychoactive ∆^9^-tetrahydrocannabivarin (∆^9^-THCV), cannabidivarin (CBDV), and ∆^9^-tetrahydrocannabinolic acid (Δ^9^-THCA). ∆^9^-THC acts as a potent partial agonist on endocannabinoid receptor CB_1_, influencing both GABAergic and glutamatergic synaptic transmission. The clinical use of medical marijuana is limited because the anticonvulsant effect of ∆^9^-THC is relatively unpredictable. It acts simultaneously on several receptor targets, such as the transient receptor potential (TRP) cation channels TRPA1, TRPV2, and TRPM8; the orphan G-coupled protein receptor GPR55; the 5-HT_3A_ receptor; the peroxisome proliferator-activated receptor gamma (PPARγ); the μ- and δ-opioid receptors, the β-adrenoreceptors; and some subtypes of Ca*^2+^,* K^+^, and Na^+^ channels. Interestingly, some experiments have shown medical marijuana to have no or even a pro-convulsant effect [104]. Δ^9^-THCA is used to prevent seizures, e.g., in the USA. This metabolic precursor of Δ^9^-THC is more affordable and should have only minor psychoactive properties [106]. Experimental observations have demonstrated that it has anticonvulsant effects via the modulation of ion channels and enzymes crucial for the biosynthesis of the endocannabinoid 2-arachidonoylglycerol [104]. The mechanisms of anticonvulsant activity of Δ^9^-THCV and CBDV are not well understood. Both compounds probably exert their anticonvulsant effects via non-CB_1_/CB_2_ mechanisms. TRPV1, TRPV2, TRPA1, and TRPM8 channels are the likely molecular targets of Δ^9^-THCV and CBDV [107].

CBD is the most promising of these agents, with effects proved by several clinical trials, especially for the treatment of drug-resistant epilepsies (see Section 5. Clinical data). Recent studies have proposed that its anticonvulsant effect may involve agonistic activity at the TRPV1 channel, the blockade of human T-type VGCCs, the modulation of various receptors such as 5-HT_1A_, 5-HT_2A_, GPR55, adenosine A1 and A2, voltage-dependent anion-selective channel protein 1 (VDAC1), or an influence on the release of TNF-α [104].

Unlike other natural constituents mentioned in this review, the metabolism, pharmacokinetics, side effects, and interactions of the main phytocannabinoids have been and continue to be deeply studied. Smoked, inhaled, or vaporized Δ^9^-THC has a bioavailability in the range from ~10–35% in contrast to (~6%) by oral administration. CBD and CBDV are hardly soluble in water, and their bioavailability after oral administration is poor, but many trials have evaluated cannabinoids suspended in sesame oil or mixed with glycocholate to increase their bioavailability, and intranasal, sublingual, and transdermal applications are common. Unfortunately, because of their lipophilic properties, large amounts of cannabinoids accumulate in adipose and other tissues, especially with repeated administration. Negative interactions with other drugs metabolized by the cytochrome P450 system or isoenzymes CYP3A4, CYP2C19, CYP2C9, and CYP2D6 should be taken into account, especially in Europe, where the co-administration of CBD and clobazam has been approved as adjunctive treatment of Dravet syndrome (DS) and Lennox–Gastaut syndrome (LGS). The strong inhibition of CYP2C19 by CBD leads to a remarkable rise in clobazam concentration, which may contribute to side effects, including somnolence and sedation. Finally, potential pharmacodynamic interactions with conventional antiepileptics must be investigated as well [104,108].

## 5. Clinical Data

Although many natural compounds exert significant anticonvulsant activity in vivo, clinical data from human subjects are almost nonexistent. Some timid attempts have been made; but a robust, double-blind, placebo-controlled trial is missing in most cases.

We cannot begin a discussion of clinical data with anything other than medicinal cannabis. Many clinical trials have reported anticonvulsant effects of constituents isolated from *C. sativa* and medicinal cannabis itself. Although recent studies describe the safety and efficacy profiles of medicinal cannabis and CBD as comparable, the potential use of cannabis extract is not recommended because of adverse events related to THC, especially in long-term use [109]. Therefore, a highly purified CBD (Epidiolex^®^) remains the only cannabis-based product approved in 2018 by the Food and Drug Administration (FDA) for the treatment of drug-resistant epilepsies (LGS and DS). The European Medicine Agency (EMA) followed in 2019 by approving CBD (Epidyolex^®^) in combination with clobazam as adjuvant therapy for LGS and DS [108].

Epidiolex (100 mg/mL CBD solution in sesame oil) was approved based on results reported by Devinsky and coworkers. An open-label interventional trial including 214 patients (aged 1–30 years) with severe, intractable, childhood-onset, treatment-resistant epilepsy was undertaken at 11 epilepsy centers across the USA. CBD reduced seizure frequency and appeared to be an effective treatment option in highly treatment-resistant epilepsies, such as DS or LGS [110]. A double-blind, placebo-controlled trial on 225 participants (aged 2–55 years) investigated the effect of CBD on drop seizures in LGS. The administration of CBD at a dose of 10 or 20 mg/kg/day and added to a conventional therapy decreased the frequency of seizures compared to a placebo [111]. Similarly, a reduction in convulsive seizure frequency was achieved in a randomized, double-blind, placebo-controlled study that evaluated the effect of CBD on 120 participants (aged 2–18 years) with DS [112]. Recently, an open-label extension trial showed that long-term use of add-on CBD had an acceptable safety profile and led to sustained reductions in seizure frequency in patients with treatment-resistant DS [113]. However, as the clinical impact of CBD is difficult to predict, the individual’s response should be carefully observed [114].

Cannabidivarin (CBDV), a natural *n*-propyl analog of CBD, has been tested in a phase II clinical trial with a double-blind, randomized, placebo-controlled design, involving 162 adult patients (aged 18–65 years). The study was intended to evaluate the anticonvulsant effect of CBDV as add-on therapy in drug-resistant focal seizures [115], but the results of the study have not been published, and in 2018, GW Pharmaceuticals announced that the trial did not meet its primary endpoint, i.e., the percent change in focal seizure frequency from baseline to the end of treatment in subjects taking CBDV compared with a placebo [108,115].

Although in previous parts we have focused only on pure and structurally characterized compounds, black seed oil, the main product obtained from *Nigella sativa*, should not be omitted. A prospective, randomized, single-blinded, crossover pilot study has examined an adjuvant therapy with black seed oil on intractable pediatric seizures. Unfortunately, the administration of 40–80 mg/kg/day of black seed oil for a 4-week period showed a statistically non-significant effect on seizure frequency and serum levels of oxidative stress parameters [116]. Nevertheless, the preclinical findings of the significant anticonvulsant activity of thymoquinone, the major monoterpene of black seed oil, led to a pilot study that involved 22 juvenile patients with refractory epilepsy. After four weeks of administration of thymoquinone at a dose of 1 mg/kg/day, patients exhibited a significantly reduced frequency of seizures for thymoquinone compared with placebo groups. As most patients were taking other AEDs, the influence of thymoquinone on the blood levels of these drugs cannot be ruled out. Altogether, thymoquinone was generally well-tolerated with no serious adverse effects, such as nausea or somnolence [53].

Several alkaloids have also been tested in a clinical setup, with huperzine A (HupA) showing the most promising results. HupA is a well-known acetylcholinesterase (AChE) inhibitor that exerts anticonvulsant activity by activating cortical GABA transmission [66]. Although it has been demonstrated that naturally occurring (−)-HupA inhibited AChE 38-fold more potently than its synthetic (+)-isomer [117], (−)- and (+)-HupA both blocked the NMDA channel similarly [99]. Clinical testing of HupA has determined that the immediate-release formulation currently available is not adequate for clinical development as an antiseizure medication because its cholinergic side effects (nausea and vomiting) limit the acceptable dose. Attention was therefore focused on the development of an extended-release (ER) formulation of HupA to reduce dosing and side effects [118]. A phase I clinical trial to evaluate the bioavailability, safety, and tolerability of an ER formulation of HupA has recently been completed. The study was conducted on eight participants who were administered an initial dose of 0.5 mg twice daily (b.i.d.). The dose was increased every 2–3 days up to the maximum tolerated dose of 2.5 mg twice daily (b.i.d.) (NCT03156439). A clinical study (involving sixteen participants) is currently underway to evaluate an ER formulation of HupA for the treatment of adult focal impaired awareness seizures (NCT03474770). Since HupA is otherwise well-tolerated, shows no serious adverse effects, and has favorable pharmacokinetic properties [119], it is worth watching whether or not this substance will find application in clinical practice.

Reports of beneficial effects of quinidine in patients with *KCNT1* mutations are increasing dramatically, supporting the use of quinidine in the treatment of epilepsy of infancy with migrating focal seizures (EIMFS), which is also known as migrating partial seizures of infancy (MPSI). The *KCNT1* gene encodes the sodium-dependent potassium channel, with quinidine being a partial antagonist of the *KCNT1* channel [120]. Quinidine has been reported to decrease seizure frequency in one patient with the Y796H mutation in the *KCNT1* gene, but not in another patient with the K629N mutation in the same gene [121]. Other studies indicate that quinidine could be a candidate drug for the treatment of *KCNT1*-positive epilepsies [120,122,123] even though its efficacy has been demonstrated in only a small number of patients. Abdelnour et al. suggest that response may be age-dependent because, in their report, only the 3-month-old infant responded to quinidine, while two older children (9 and 13 years) did not [124]. On the other hand, quinidine did not show efficacy in six patients with autosomal dominant nocturnal frontal lobe epilepsy (ADNFLE) due to *KCNT1* mutations in a single-center, inpatient, order-randomized, blinded, placebo-controlled, crossover trial [125]. Similarly, an observational study of 43 patients to evaluate the treatment responsiveness of patients with *KCNT1*-related epilepsy reported that quinidine was not utilized in any patients with an ADNFLE phenotype, whereas quinidine treatment was attempted in 17 patients with an EIMFS phenotype. Unfortunately, a reduction > 50% in seizures was seen in only four patients treated with quinidine [126]. Despite its controversial efficacy, quinidine deserves attention and should be evaluated further because too few cases have been studied.

## 6. Conclusions

This review describes the significant anticonvulsant effects of various natural products. Although their anticonvulsant activity is evident in in vivo models of epilepsy, and in most cases, their molecular targets are clearly recognized, not many plant-derived compounds have made the step up to evaluation by clinical trials. As summarized in Table 1, Table 2 and Table 3 and Tables S1–S3 [127,128,129,130,131,132,133,134,135,136,137,138,139,140,141,142,143,144,145,146,147,148,149,150,151,152,153,154,155,156,157,158,159,160,161,162,163,164,165,166,167,168,169,170,171,172,173,174,175,176,177,178,179,180], natural anticonvulsants can be found across the plant kingdom. They affect the same targets as conventional AEDs yet are still overlooked within drug development. An argument based purely on limited solubility and bioavailability becomes less and less acceptable in an era of nanocarriers and bioenhancers. Natural products can also serve as suitable templates for semisynthetic derivatives with better efficacy and pharmacokinetic properties. Moreover, plant-based therapy is usually well-tolerated, and in most cases, it is associated with no or only moderate adverse effects (if used sensibly). Plant-based therapy has a number of benefits. Given the multiple effects of many natural compounds, including neuroprotective and anti-inflammatory activity, plant-based therapy could become a powerful anticonvulsant tool and should not be overlooked. This review assumes that soon Epidiolex will not be the only plant-derived product approved for the treatment of epilepsy-related disorders.

## Data Availability

Data is contained within the article and supplementary material.

## Supplementary Materials

The following supporting information can be downloaded at: https://www.mdpi.com/article/10.3390/ph16081061/s1, Table S1: Natural products with other targets title; Table S2: Unknown targets; Table S3: Natural products with potential anti-epileptic activity via neuroprotection and anti-inflammatory action; References for Supplementary Material.
