# Supplementary material for: Treating Epilepsy with Natural Products: Nonsense or Possibility?
Source: Pharmaceuticals (Basel). 2023 Jul 26;16(8):1061. doi: 10.3390/ph16081061 (PMC10459181; doi:10.3390/ph16081061)
Supplement: Supplementary file 1 [file pharmaceuticals-16-01061-s001.zip › pharmaceuticals-2462143-supplementary.pdf]

Table S1: Natural products with other targets.

Compound	Effective dose	Animal model	Seizure-inducing agent	Mechanism	Source
Oleuropein<br>(secoiridoid)	10, 20, and 30 mg/kg<br>i.p.	Male NMRI mice	PTZ	Possibly *via* opioidergic/nitrergic pathways	[127]
Tanshinone IIA<br>(diterpene)	59 mg/kg of larvae<br>0.5–1 mg/kg mouse<br>i.v.	Zebrafish larvae<br>Male NMRI mice	PTZ<br>6-Hz test	Reduction of *c-fos* expression	[128]
Curcumin<br>(phenolic)	30 mg/kg i.p.	Wild-type Std-ddY<br>male mice	KA	↓ in bulk histone H4 acetylation<br>↓ in *c-fos* and *c-jun* mRNA expression	[129]
	1500 ppm w/w	Male Wistar rats	Ferric chloride	Antioxidant activity<br>Binding Fe^{2+}/Fe^{3+} ions<br>Amelioration of membrane fluidity<br>Inhibition of cytosolic PKC activity- ↓ PKC-mediated excitotoxicity<br>↑ Na^{+}-K^{+} ATPase activity<br>Neuroprotective activity in cortex	[130]
Resveratrol<br>(stilbenoid)	20, 40, and 80 mg/kg<br>RE (40 mg/kg i.p.) +<br>sodium valproate<br>(150 mg/kg)<br>RE+DZP (2 mg/kg)	Male Wistar rats	PTZ	Adenosinergic activity	[131]
Berberine<br>(alkaloid)	25, 50, and 100<br>mg/kg/day i.g.<br>(for 7 days)	Male Sprague-Dawley rats	Pilocarpine	Anticonvulsant and neuroprotective effect,<br>block of potassium currents	[132]
	10 and 20 mg/kg i.p.	Male Swiss mice	PTZ, MES, KA	Modulation of neurotransmitter systems	[133]
Harmane<br>(alkaloid)	2.5, 5, and 10 mg/kg<br>i.p.	Male and female<br>Swiss mice	MES<br>PTZ	Interaction with imidazoline and<br>benzodiazepine receptors	[134]
Mesaconitine<br>(alkaloid)	0.003–1 μM	Hippocampal slices<br>of male Wistar rats	Low Mg^{2+}/high K^{+}-ACSF<br>Bicuculline	Activation of α-adrenoceptors	[135]
Piperine<br>(alkaloid)	40 and 80 mg/kg i.p.	Male KM mice	MES<br>PTZ	Probably TRPV1 receptor agonist	[136]

Quinine (alkaloid)	60 mg/kg i.p.	Male BALB/c mice	PTZ	Blockade of Cx36 channels	[137]
	200, 400, and 1000 nmol i.c.v.	Male Wistar rats	Penicillin	Blockade of Cx36 channels	[138]
Sanjoinine A (alkaloid)	4 and 8 mg/kg p.o.	Male ICR mice<br>Male Sprague-Dawley rats	KA	Increasing intracellular Cl^- and reducing intracellular Ca^{2+} levels	[139]
Tetrahydropalmatine (alkaloid)	10 or 15 mg/kg i.p.	Male Sprague-Dawley rats	PTX	Inhibition of amygdaloid dopamine release	[140]

Table S2: Unknown targets.

**Compound**	**Effective dose**	**Animal model**	**Seizure-inducing agent**	**Mechanism**	**Source**
β-caryophyllene (sesquiterpene)	100 mg/kg i.p.	C57BL/6 mice	PTZ	Unknown	[141]
	30 mg/kg i.p.<br>50 and 100 mg/kg i.p.	Male ICR mice	MES<br>KA	CB2 receptor agonist	[142]
Valerenic acid (sesquiterpene)	37 µg/mL	Adult zebrafish	PTZ	Unknown (possibly modulation of GABA receptors)	[143]
Abietic acid (diterpene)	30 and 100 mg/kg p.o., i.p.	Swiss mice	MES	Unknown	[144]
Phytol (diterpene)	25, 50, and 75 mg/kg i.p.	Male Swiss mice	Pilocarpine	Unknown	[145]
Caudatin-3-*O*-*β*-D-Cym-(1→4)-*β*-D-Cym-(1→4)-*β*-D-Cym (steroid)	10 µg/mL	Zebrafish larvae	PTZ	Unknown	[146]
Caudatin-3-*O*-*β*-D-Ole-(1→4)-*β*-D-Ole-(1→4)-*β*-D-Cym-(1→4)-*β*-D-Cym (steroid)	10 µg/mL	Zebrafish larvae	PTZ	Unknown	[146]

Cyanoauriculoside G (steroid)	100 mg/kg p.o. (ED_{50} = 88.1 mg/kg)	Male KM mice	MES	Unknown	[147]
Cynawilfoside A (steroid)	100 mg/kg p.o. (ED_{50} = 48.5 mg/kg)	Male KM mice	MES	Unknown	[147]
Cynauricoside A (steroid)	100 mg/kg p.o. (ED_{50} = 95.3 mg/kg)	Male KM mice	MES	Unknown	[147]
Otophylloside B (steroid)	10 µg/mL	Zebrafish larvae	PTZ	Unknown	[146]
	100 and 200 µM	Zebrafish larvae	PTZ	Unknown	[148]
Otophylloside F (steroid)	300 µM	Zebrafish larvae	PTZ	Unknown	[148]
Rostratamine 3-*O*-*β*-D-Ole-(1→4)-*β*-D-Cym-(1→4)-*β*-D-Cym (steroid)	100 and 200 µM	Zebrafish larvae	PTZ	Unknown	[148]
Torvoside J (steroid)	140 µM	Zebrafish larvae *Xenopus laevis* oocytes	PTZ	Unknown (weak $GABA_A$ modulation)	[149]
Wilfoside C1N (steroid)	100 mg/kg p.o. (ED_{50} = 124.1 mg/kg)	Male KM mice	MES	Unknown	[147]
Wilfoside K1N (steroid)	100 mg/kg p.o. (ED_{50} = 72.3 mg/kg)	Male KM mice	MES	Unknown	[147]
Ursolic acid (triterpene)	2.3 mg/kg i.g.	Male Swiss mice	PTZ	Unknown	[150]
	50 and 100 mg/kg i.p.	Naïve male Swiss mice	6-Hz test MES	Unknown	[151]
	10, 30, and 100 mg/kg i.p.	Male Swiss mice	PTZ	Modulation of $GABA_A$ receptor	[152]
Decursin (coumarin)	20 mg/kg i.p.	Male C57BL/6 mice	KA	Protective effect against glutamate-induced neurotoxicity	[153]
Acacetin (flavonoid)	10 and 20 mg/kg/day p.o. (for 2 weeks)	ICR male mice	PTZ	Unknown	[154]

				(possibly modulation of GABA, opioid, and serotonin receptors)	
Apigenin (flavonoid)	25 and 50 mg/kg i.p.	Male Sprague-Dawley rats	PTX	Unknown (possibly modulation of GABA receptors)	[155]
Goodyerin (flavonoid)	25 and 50 mg/kg i.p.	Male ICR mice	PTX	Unknown (possibly modulation of GABA receptors)	[156]
Kaempferitrin (flavonoid)	1 µg/µL i.c.v.	Male Swiss Webster mice	PTZ	Unknown (agonistic 5-HT_{1A} activity)	[157]
Linarin (flavonoid)	10 and 20 mg/kg/day p.o. (for 2 weeks)	ICR male mice	PTZ	Unknown	[154]
Naringenin-7-*O*-methyl ether (flavonoid)	25 µM	Zebrafish larvae	PTZ	Unknown	[158]
Naringenin-4′,7-*O*-dimethyl ether (flavonoid)	12.5 µM<br>12.5 mg/kg i.p.<br>6.25 mg/kg i.p.	Zebrafish larvae<br>Male C57BL/6 mice<br>Male NMRI mice	PTZ<br>6-Hz test	Unknown	[158]
Erysothrine (alkaloid)	0.125–3 µg/µL i.c.v.	Male Wistar rats	Bicuculline, PTZ, NMDA, KA	Unknown	[159]
TMC-120A (alkaloid)	20 µg/µL<br>1.25, 2.5, and 10 mg/kg i.p.	Zebrafish larvae<br>Male NMRI mice	PTZ<br>6-Hz test	Unknown	[160]
TMC-120B (alkaloid)	20 µg/µL<br>10 mg/kg i.p.	Zebrafish larvae<br>Male NMRI mice	PTZ<br>6-Hz test	Unknown	[160]
TMC-120C (alkaloid)	20 µg/µL	Zebrafish larvae	PTZ	Unknown	[160]
Penicisochroman G (alkaloid)	12.5 µg/µL	Zebrafish larvae	PTZ	Unknown	[160]
Ustusorane B (alkaloid)	12.5 µg/µL	Zebrafish larvae	PTZ	Unknown	[160]
Pseurotin A_2 (γ-lactams)	12.5 µg/µL<br>40 mg/kg i.p.	Zebrafish larvae<br>Male NMRI mice	PTZ<br>6-Hz test	Unknown	[161]
Azaspirofuran A (γ-lactams)	12.5 µg/µL<br>40 mg/kg i.p.	Zebrafish larvae<br>Male NMRI mice	PTZ<br>6-Hz test	Unknown	[161]

Table S3. Natural products with potential anti-epileptic activity *via* neuroprotection and anti-inflammatory action.

**Compound**	**Effective dose**	**Animal model**	**Seizure-inducing agent**	**Mechanism**	**Source**
Borneol (monoterpene)	25 mg/kg i.p.	Male Swiss mice	PTZ	Neuroprotective effect	[162]
Thymoquinone (monoterpene)	10 mg/kg i.p., twice	Male Sprague-Dawley rats	Lithium-Pilocarpine model of SE	Protective effect *via* downregulation of TNF-α and COX-2 in the brain	[56]
ar-Turmerone (sesquiterpene)	50 mg/kg i.p.<br>46 µM	Male C57BL/6 mice<br>Zebrafish larvae	PTZ<br>6-Hz test	Downregulation of *c-fos* (upregulation of BDNF)	[163]
Bilobalide (sesquiterpene)	5 and 10 mg/kg i.p.	Male Sprague-Dawley rats	Middle cerebral artery occlusion	Neuroprotective effect (↓ TNF-α, IL-1β, p-JNK1/2, and p-p38 MAPK expression)	[62]
Asiatic acid (triterpene)	40 mg/kg/day p.o. (for 7 days)	Male C57BL/6 mice	KA	Neuroprotective effect (↓ IL-1β, TNF-α, PGE_2, COX-2, and Bax mRNA expression)	[164]
	5–30 µM	Hippocampal terminals of male Sprague-Dawley rats	4-AP	Attenuation of the calcium influx via N- and P/Q-type calcium channels, subsequently decreasing glutamate release	[165]
Maslinic acid (triterpene)	40 mg/kg/day p.o. (for 7 days)	Male C57BL/6 mice	KA	Neuroprotective effect (↓ IL-1β, TNF-α, PGE_2, COX-2, and Bax mRNA expression)	[164]
Otophylloside N (steroid)	5 and 10 µg/mL<br>2.5 and 5 µg/kg i.p. (t.i.d. for 7 days)<br>12, 25, and 50 µg/mL	Primary cortical neurons<br>C57BL/6J mice<br>Zebrafish larvae	PTZ	Upregulation of Bax/Bcl-2 and downregulation of *c-fos* expression	[166]
16-*O*-Acetyl digitoxigenin (steroid)	1.8 mg/kg/day i.p. (for 4 weeks)	Hippocampal neurons of male ICR mice	Pilocarpine	Inhibition of activation of p-mTOR/p-70S6K pathway, inhibition of mTOR signaling pathway, neuroprotective effect	[167]
Corilagin (tannin)	10 and 20 mg/kg i.p. (for 24 days)	Male Wistar rats	PTZ	Anti-inflammatory (↓ TNF-α, ↑ IL-10)<br>Antioxidant (↓ ROS production, ↓ CAI activity)	[168]
Gastrodin (phenolic)	PHT:GD = 1:50 (8.59:429.27 mg/kg)	Male KM mice	Penicillin	Neuroprotective<br>↑ NF-κB for induction of synaptic plasticity	[169]

Arbutin (phenolic)	25 and 50 mg/kg i.p. (for 10 days)	Male NMRI mice	PTZ	Neuroprotective effect (↓ TNF-α, IL-6, and GFAP mRNA expression)	[170]
Vanillyl alcohol (phenolic)	100 mg/kg i.p.	Male Sprague-Dawley Rats	Ferric chloride	Antioxidant activity (↓ lipid peroxide levels)	[171]
Resveratrol (stilbenoid)	20 and 40 mg/kg i.p., three times	Wistar rats of either sex	KA	Antioxidant activity	[172]
	20 mg/kg in liposome i.v.	Male Sprague-Dawley rats	Penicillin	Antioxidant activity (↑ in GST and SOD activity, ↑ GSH and ↓ MDA levels)	[173]
Baicalin (flavonoid)	100 mg/kg i.p.	Male Sprague-Dawley rats	Pilocarpine	Neuroprotective (↓ lipid peroxidation, ↓ NO, ↑ GSH, protects against neuronal apoptosis)	[174]
Chrysin (flavonoid)	10 µM	BV-2 mouse microglial cell line		Neuroprotective (↓ iNOS, COX-2 expression → ↓ NO, ↓ TNF-α, ↓ IL-1β) Inhibition of the activation of JNK and NF-κB	[175]
Fisetin (flavonoid)	10 and 25 mg/kg i.p.	Male Swiss mice	PTZ, STR, INH, MES	↑ GABA Antioxidant activity (↓ NO, ↓ XO)	[176]
	20 mg/kg/day p.o. (for 12 weeks)	Male Wistar rats	$FeCl_3$	Antioxidant activity (↓ lipid peroxidation) Restore the activity of the Na^+, K^+-ATPase	[177]
Luteolin (flavonoid)	10 and 20 mg/kg i.p.	Swiss albino mice	PTZ	Antioxidant activity (↑ GSH and ↓ MDA)	[178]
Naringin (flavonoid)	40 and 80 mg/kg i.p., (for 7 days)	Male Wistar rats	KA	Neuroprotective and antioxidant (↓ MDA, ↑ GSH, ↓ TNF-α)	[179]
Berberine (alkaloid)	25, 50, and 100 mg/kg i.g. (for 7 days)	Sprague-Dawley rats	Pilocarpine	Anticonvulsant and neuroprotective effect, block of potassium currents	[180]
Sinomenine (alkaloid)	20, 40, and 80 mg/kg i.p.	Male Sprague-Dawley rats	PTZ	Neuroprotective and anti-inflammatory effect, attenuation of IL-1β, IL-18, IL-6, TNF-α	[180]
